# The T Cell Repertoires from Nickel Sensitized Joint Implant Failure Patients

**DOI:** 10.3390/ijms22052428

**Published:** 2021-02-28

**Authors:** Lan Chen, Yan Zhang, Karin Pacheco, Shaodong Dai

**Affiliations:** 1 Skaggs School of Pharmacy and Pharmaceutical Sciences, University of Colorado Anschutz Medical Campus, Aurora, CO 80045, USA; lan.chen@cuanschutz.edu (L.C.); yan.zhang@cuanschutz.edu (Y.Z.); 2 Department of Medicine, National Jewish Health, Denver, CO 80206, USA; PachecoK@NJHealth.org

**Keywords:** metal hapten, hypersensitivity, single-cell sequencing, joint implant failure, TCR repertoire

## Abstract

Nickel (Ni^2+^) is one of the most common allergens, affecting around 10–15% of the general population. As the demand for orthopedic implant surgery rises, the number of surgical revisions due to joint implant failure also increases. There is evidence that some patients develop joint failure due to an immune response to a component of the implant, and we have found that Ni^2+^ is an especially important cause. Hence, understanding the mechanisms by which Ni^2+^ allergy induces joint implant failure becomes a critical research question. The structural basis of Ni^2+^ activation of pathogenic T cells is still not clear. The purpose of this study was to characterize Ni^2+^-reactive T cell repertoires derived from the peripheral blood of joint failure patients due to Ni^2+^ sensitization using single-cell sequencing techniques. We stimulated the proliferation of Ni^2+^ -reactive T cells from two implant failure patients in vitro, and sorted them for single-cell VDJ sequencing (10× genomics). We identified 2650 productive V-J spanning pairs. Both TCR α chains and β chains were enriched. TRBV18 usage is the highest in the P7 CD4+ population (18.1%), and TRBV5-1 usage is the highest in the P7 CD8+ population (12.1%). TRBV19 and TRBV20-1 segments are present in a high percentage of both P7 and P9 sequenced T cells. Remarkably, the alpha and beta chain combination of TRAV41-TRBV18 accounts for 13.5% of the CD4+ population of P7 patient. Compared to current Ni specific T cell repertoire studies of contact dermatitis, the Vα and Vβ usages of these joint implant failure patients were different. This could be due to the different availability of self-peptides in these two different tissues. However, TRBV19 (Vβ17) was among frequently used TCR β chains, which are common in previous reports. This implies that some pathogenic T cells could be similar in Ni^2+^ hypersensitivities in skin and joints. The alignment of the TCR CDR3β sequences showed a conserved glutamic acid (Glu) that could potentially interact with Ni^2+^. The study of these Ni^2+^ specific TCRs may shed light on the molecular mechanism of T cell activation by low molecular weight chemical haptens.

## 1. Introduction

There were approximately 5.2 million total knee arthroplasties performed in the United States during the 2000–2010 period [1], and the demand for total hip and knee arthroplasties is predicted to grow to 850,000 and 1.91 million, respectively, by 2030 [2]. Total joint replacement constitutes the highest cost for Medicare programs, and similar demand is growing worldwide [3,4]. The majority of joint replacements are performed for osteoarthritis, increasingly common in an aging world population, with a history of sports induced injuries and rising rates of obesity. Other, less common causes include connective tissue disease destruction of large joints, avascular necrosis caused by injury to vascular joint structures, and treatment with steroids, with smaller contributions from fractures, benign and malignant bone tumors, and hemarthrosis (https://orthoinfo.aaos.org/en/treatment/total-joint-replacement/).

Joint replacement prosthetic manufacturing is dominated by a few large companies, although there are smaller companies providing specialized implants. The majority of commercially available implants are composed of a cobalt/chromium alloy containing 60% cobalt, 30% chromium, 5% molybdenum, with from 0.5 to 2% nickel. Stainless steel, commonly used in orthopedic screws and staples, contains from 13 to 15.5% nickel, from 12 to 18% chromium, from 0.8 to 1.8% manganese, and the balance of iron. All these metals, with the exception of iron, can cause sensitization and disease in humans, most frequently nickel, cobalt, and chromium. The most dominant of these is nickel. 

Generally, up to 90% of joint replacements do well, and provide significant health and socioeconomic benefits to the recipients. About 10% of these, however, will fail, due to infection, mechanical issues, and immune mediated responses to the implant [5]. All metal implants undergo corrosion in synovial fluid, and release metal ions that can act as haptens to activate the immune system [6]. As the number of arthroplasties increases worldwide, the incidence of implant failure caused by Ni^2+^ hypersensitivity reactions will grow concomitantly.

Long term sensitization to Ni^2+^ is known to be a type IV cell-mediated hypersensitivity. This process can be induced by Ni^2+^ binding to the TLR4 receptor [7] on antigen presenting cells (APCs) such as dendritic cells, activating them, and eliciting the secretion of proinflammatory cytokines. APCs then carry the Ni^2+^ antigen to regional lymph nodes, where they prime naive T lymphocytes to differentiate into effector T cells, and begin the process of antigen-specific T cell activation [8]. αβ T cells commonly recognize peptide antigens bound to MHCI and MHCII molecules, and play a central role in adaptive immunity. MHC molecules can also present small molecules in addition to peptides to T cells. However, the specific molecular basis of T cell activation by Ni^2+^ ions still remains unclear.

Although studies of joint implant failure have established the association with Ni^2+^ hypersensitivity, the dominant TCRs responsible for the process are still unknown. Most previous studies of the molecular mechanisms of Ni^2+^ hypersensitivity have mainly focused on Ni^2+^-reactive T cells from contact dermatitis patients. Here, the antigen presenting cells are Langerhans cells and dermal dendritic cells, which differ from the macrophages and dendritic cells relevant in the joint, such that the Ni^2+^ response in the joint could be totally different from the dermal response [9,10]. 

In contact dermatitis subjects, isolation of a panel of Ni^2+^-specific T cell clones from peripheral blood mononuclear cells (PBMCs) by the Weltzien group identified that TRBV19 (Vβ17) TCRs predominated [11,12]. One of the TRBV19 CD4+ TCR, SE9, was MHC and peptide promiscuous [13]. The other TRBV19 TCR, ANi2.3, was strictly DR52c restricted, requiring an unknown endogenous peptide to effectively present Ni^2+^ ions [14]. We identified a panel of Ni^2+^ independent mimotopes that can directly activate the ANi2.3 TCR independent of Ni^2+^ [15]. The ternary structure of the ANi2.3 TCR, MHC and mimotope confirmed that Ni^2+^ presentation required both a specific MHC and peptides [15]. Recently, a study by Bechara et al. also showed the dominant usage of TRBV19 in highly expanded clones from Ni^2+^-specific naive T cell lines derived from Ni^2+^ sensitized contact dermatitis subjects [16]. However, another paper found dominant usage of TRAV9-2 rather than TRBV19 in CD154 positive nickel-stimulated T cells [17]. The ternary complex structure of the ANi2.3 TCR, DR52c and Ni^2+^ dependent mimotope was determined. However, it is still not clear as to how the unknown endogenous peptide presents Ni^2+^ to the ANi2.3 TCR, or whether the TRBV19 TCR is the dominant TCR that also induces joint implant failure due to Ni^2+^ sensitization. We previously found that around 1% of peripheral blood T cells from several Ni-sensitized joint failure patients interact with the DR52c-mimotope. In addition, the percentage of TRBV19 CD4 T cells increases from 0.76% to 13.3% when stimulated with Ni^2+^ in vitro [18]. This suggests that the TRBV19 TCR may also play an important role in inducing Ni^2+^ sensitization in periprosthetic tissue after arthroplasty.

In this study, a 10× single cell VDJ sequencing technique was utilized to analyze the Ni^2+^-reactive T cell repertoire derived from the PBMCs from two joint failure patients. We identified several highly expanded T cell clones that preferentially used specific TCR alpha and beta chains. Furthermore, the alignment of the CDR3β sequences of these TCRs shows a conserved Glu at the last fourth position that could directly interact with Ni^2+^ ions. Given the rising number of joint replacements using nickel containing implants world-wide, and the concomitant rise in failure due to nickel sensitization, these results provide an important insight in this process, as well as suggestions for therapeutic interventions.

## 2. Results

### 2.1. Characterization of Subject Clinical Features

Out of a population of *n* = 483 implant failure patients, 29% were sensitized to Ni^2+^, peripheral blood was selected from two female joint implant failure patients, designated P7 and P9. Both patients were post implant: P7 with a left total hip arthroplasty with a mixed, CoCrMo and Titanium alloy implant; P9 had a right total knee arthroplasty with a CoCrMo alloy implant and bone cement. Both patients were Ni^2+^ sensitized based on patch testing, along with a strongly positive Ni^2+^ lymphocyte proliferation test (NiLPT) stimulation index. More importantly, when stimulated with Ni^2+^, both PBMCs demonstrated a clear population that stained with a tetramer of the DR52c/Ni^2+^ mimotope [10]. The clinical data summary is shown in Table 1. PBMCs were separated from fresh blood and stored in liquid nitrogen. DR genes were identified by high resolution methods: P7 is positive for DR4, DR7, and DR53, whereas P9 is positive for DR7, DR11, DR53, and DR52b.

### 2.2. PBMCs Proliferated and Secreted IFNγ with Ni^2+^ Stimulation In Vitro

**Figure 1:**
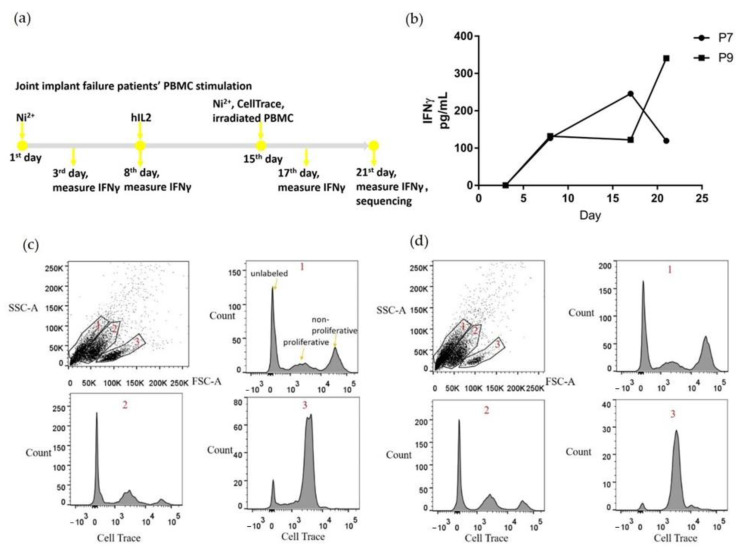
Ni^2+^ treated PBMCs include proliferating cells after three weeks in vitro culture. (**a**) shows the stimulation protocol for PBMCs derived from joint implant failure patients; (**b**) levels of secreted IFNγ in the culture media; (**c**) and (**d**) flow cytometry results of the harvested PBMCs at the last day of stimulation for P7 and P9, respectively. Based on the size and scattering, cells were separated into three populations. We examined the CellTrace dye fluorescence for each of the three populations separately. The “unlabeled”, “proliferative”, and “non-proliferative” peaks are indicated in (**c**) 1 population, and similar in the other panels. The proliferative cells in populations 2 and 3 were collected for single cell sequencing.

PBMCs from P7 and P9 patients were stimulated with Ni^2+^ and cultured for three weeks in vitro (Figure 1a). During the first week, PBMCs were cultured in media with 100 μM NiSO_4_ to support Ni^2+^-specific T cell proliferation. During the second week, the media was changed to Ni-free, and hIL2 was added to maintain cell viability. During the third week, media was changed to hIL2 free, with the addition of 100 μM NiSO_4_ and irradiated PBMCs as APCs were added to encourage proliferation of Ni^2+^-specific T cells. Cells were labeled with CellTrace proliferation dye at the beginning of the third week, to enable separation of the proliferating cells based on the fluorescence at the end of the experiment. To monitor T cell stimulation, culture supernatant was collected every three or four days for IFNγ measurement. IFNγ was selected as a proinflammatory cytokine marker that is pivotal in antiviral activity and autoinflammatory diseases, and is usually secreted by differentiated Th1 CD4+ and Tc1 CD8+ cells. Both samples demonstrated a trend of increased IFNγ secretion (Figure 1b), although P7 showed a fall in IFNγ from the 17th to the 21st day.

At the end of the 3-week stimulation, all cells were harvested, and proliferating cells were separated based on incorporation of the CellTrace proliferation dye (Figure 1c,d). For both P7 and P9, three separate cell populations were identified based on cell size and granularity. Live and proliferating lymphocytes are located in population 3 circled in 1c and 1d. Based on their size and granularity, populations 1 and 2 contained predominantly dead or dying cells not labeled with CellTrace. The proliferating cells from populations 2 and 3 were collected, labeled with totalseqC hashtag antibodies, and sent for single cell V(D)J sequencing and single cell gene expression sequencing.

### 2.3. Preferential TCR Usage in the Repertoire of Ni^2+^-Recognizing CD4+ and CD8+ T Cells

**Figure 2:**
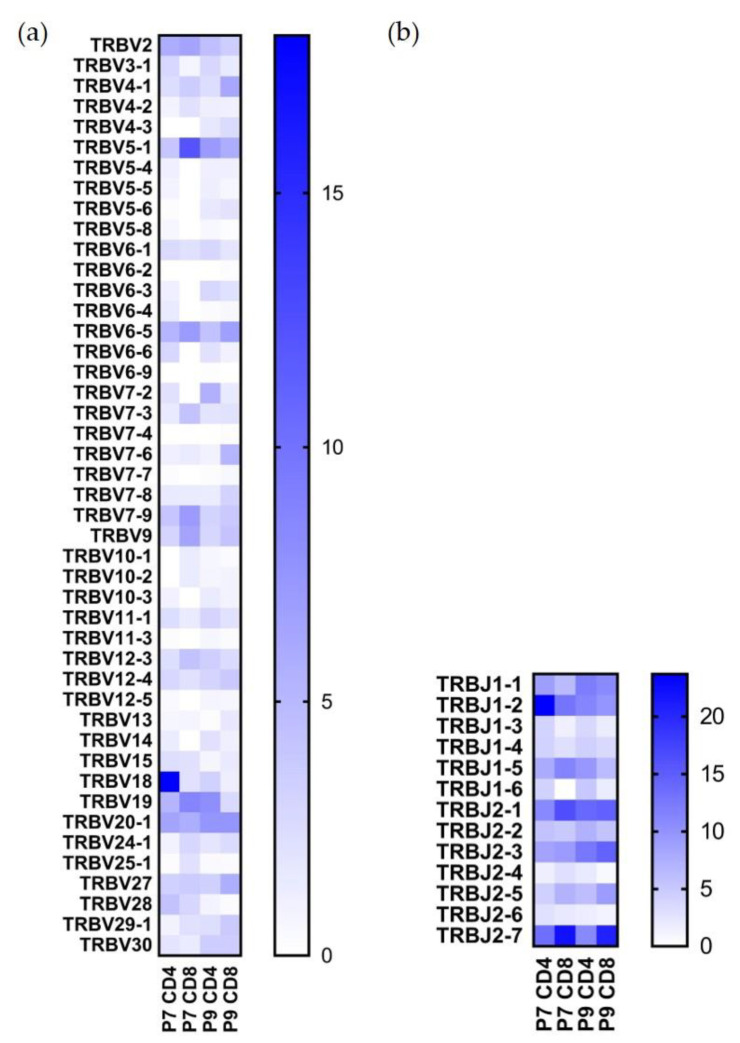
TRBV and TRBJ usage in Ni^2+^-reactive CD4+ and CD8+ T cell clones in P7 and P9 subjects. (**a**) Percentage of the TRBV gene usage. (**b**) Percentage of the TRBJ gene usage. All gene names are in IMGT nomenclature. The scale reflects the percentage present in each designated population.

**Figure 3:**
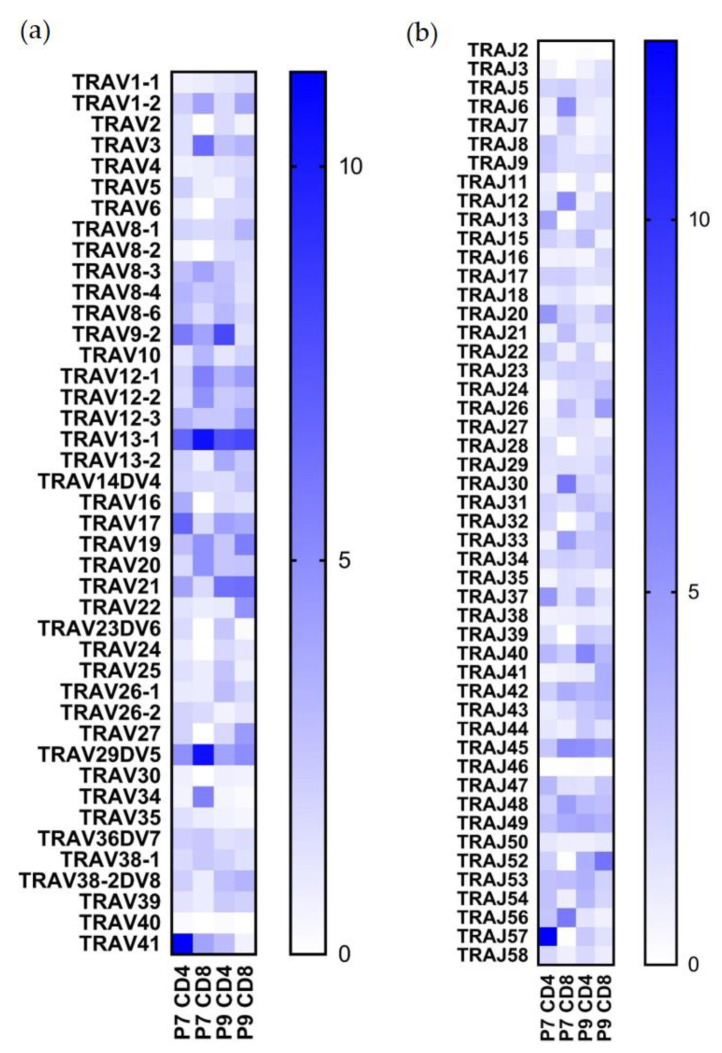
TRAV and TRAJ usage in Ni^2+^-reactive CD4+ and CD8+ T cell clones in subjects P7 and P9. (**a**) Percentage of the TRAV gene usage. (**b**) Percentage of the TRAJ gene usage. All the gene names are in IMGT nomenclature. The scale shows the percentage use in each of the four designated populations.

Single-cell VDJ sequencing yielded 3682 individual clones out of ~5000 that were sequenced, and 2650 of them included productive V-J spanning pairs. After demultiplexing hashtag results, we were able to analyze the paired TCR sequences for P7 CD4+, P7 CD8+, P9 CD4+, and P9 CD8+ cells. The TRBV and TRBJ usage percentages for these T cell types are shown in Figure 2, and the TRAV and TRAJ usage percentages are shown in Figure 3. Both TCR alpha and beta chains showed preferential usage. Noticeably, TRBV18 usage is the highest in the P7 CD4+ population (18.1%) but not in the other three populations; TRBV5-1 usage is the highest in the P7 CD8+ population (12.1%), but also found in a high percentage of the other three cell populations (Figure 2a). TRBV19 and TRBV20-1 are present in a high percentage of both P7 and P9 sequenced T cells; TRBV19 accounts for 5.3% of P7 and 8% of P9 CD4+ T cells, and TRBV20-1 accounts for 6.4% of P7 and 7.5% of P9 CD4+ T cells. As to TRBJ usage, TRBJ1-2, TRBJ2-1, and TRBJ2-7 TCR motifs are the most highly used in all four populations (Figure 2b).

As to TRAV usage (Figure 3a,b), TRAV13-1 is the most highly expanded in both CD4+ CD8+ in both patients (6.8%, 10.5%, 7.6%, and 8.1%, respectively); TRAV41 constituted 11.2% of the P7 CD4+ population but was not found in a high frequency in the other populations; TRAV29DV5 comprised 10.5% of the P7 CD8+ population; TRAJ57 was found preferentially in the P7 CD4+ population (12.4%) (Figure 3b); other TRAJ sequences were not preferentially used.

### 2.4. Highly Expanded TCR Clones Are Detected in Both CD4+ and CD8+ T Cells

The most highly expanded clones in two samples for both CD4+ and CD8+ cells are listed in Table 2. The alpha and beta chain combination of TRAV41-TRBV18 accounts for 13.5% of the P7 CD4+ population, but is not found in the other populations. Remarkably, the TRBV19 TCR accounts for 9.0% of the P9 CD4+ cells, and 1/3 of the TRBV19 T cell population is joined to the TRBJ1-5 sequence. Although Ni^2+^-reactive CD8+ T cells have not been identified in previous studies, we found some highly expanded CD8+ T cell clones for both subjects, for example, TRAV29DV5-TRBV6-5 in P7 and TRAV22-TRBV7-6 in P9.

**Figure 4:**
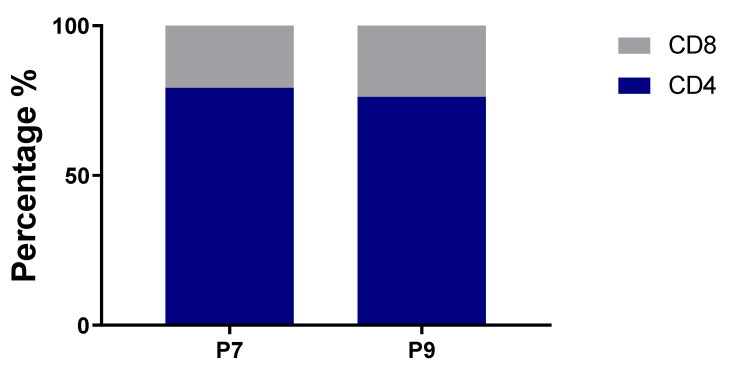
Percentage of CD4+ and CD8+ T cells in sequenced samples.

The hashtag labeling of sorted cells enabled us to calculate the relative percentages of CD4+ and CD8+ T cells for both subjects, and both have similar ratios. CD4+ and CD8+ T cells constituted 79.2% and 20.8%, respectively, for P7; and 76.2% and 23.8%, respectively, for P9 (Figure 4).

### 2.5. Conserved Glu Is Found in the CDR3 Sequences

**Figure 5:**
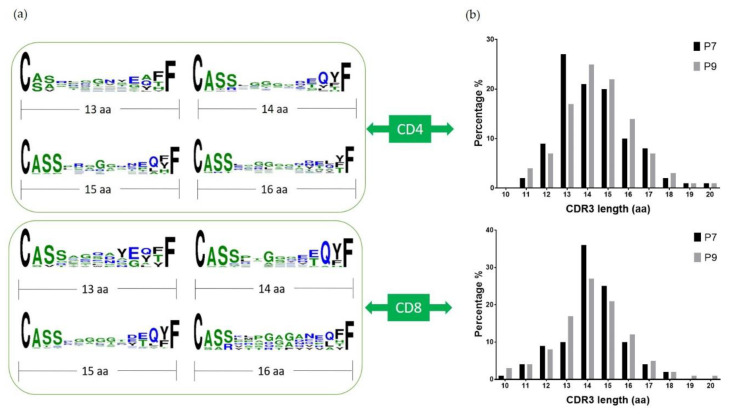
CDR3β sequence alignment and length comparison: (**a**) shows the alignment of CDR3β sequences from P7 and P9 T cells combined using Weblogo; (**b**) shows the distribution of the CDR3β length of P7 and P9 T cells, respectively, in CD4+ (top) and CD8+ (bottom) T cells. The *x*-axis is the length from amino acid C to F, and the *y*-axis is the percentage.

Previous studies have indicated that TCR beta chains have the most contact with the antigen peptide [15], and the changes in Vβ repertoire usage correspond to the Ni^2+^ response [19,20,21]. As the CDR3 region is the most variable and important for direct antigen interaction, we aligned the sequences of CDR3β from both P7 and P9 TCRs. It is noticeable that Glu is conserved at the last 4th position of CDR3 (Figure 5a) in both subjects, which could be the important amino acid to interact with the positively charged Ni^2+^. The CDR3β lengths of both P7 and P9 shared similar size distributions, and the TCR from CD4+ and CD8+ cells both peaked at 13–15 amino acid length (Figure 5b). Vβ Segments seemed to skew towards long CDR3s comparing to health controls [22]. This would suggest that CDR3 Ni^2+^ coordination would require a long flexible loop as most metal binding sites in proteins. Indeed, the CDR3β of Ni^2+^ specific TCR Ani2.3 contained 16 amino acids, and adopted a highly flexible and extended confirmation in the crystal structure [15].

## 3. Discussion

A growing number of studies demonstrate that nickel allergy not only leads to mild to severe contact dermatitis, but also contributes to joint replacement failure. Several clinical studies have documented the contribution of metal allergy, and especially nickel allergy, to joint replacement failure [5,10,23,24,25,26]. Some other clinical studies have also demonstrated improvement in nickel allergy patients once their Ni^2+^ containing implant is replaced with non-Ni^2+^ hardware, compared to those Ni^2+^ sensitized implant failure patients who are not revised and remain symptomatic [23,27]. Revision surgery for a failed implant due to sensitization is both painful and expensive, and some patients will become sensitized to the implant after the index surgery [10]. Hence, information that improves the ability to predict who will become sensitized is a valuable addition to clinical care.

Currently, information is limited as to the mechanisms leading to a Ni^2+^-induced immune response in the synovial fluid of a failed implant. Several studies have focused on the MHC–TCR interactions in Ni contact dermatitis, and the type and site of interaction may differ from the interactions leading to Ni allergy in the joint. The strength of this study lies, in part, in the selection of two patients who had undergone joint replacements with implant failure. Both had CoCrMo implants which also contain nickel. Both patients demonstrated both patch test and LPT reactivity to Ni^2+^, and we considered them ideal for studying the mechanisms of Ni-induced implant failure.

Our culture conditions also enabled us to select Ni^2+^-specific T cell clones in a more efficient way than the typical study, which requires many weeks of T cell clonal expansion and selection. In our study, we cultured PBMCs with two cycles of Ni stimulation over three weeks, interspersed with one week of hIL-2 culture to maintain cell viability, and then used the CellTrace proliferation dye to select out the proliferating cells specific for Ni. This approach allowed us to more efficiently obtain thousands of paired TCR sequences with much less time and labor. Because antigen presenting cells typically do not survive three weeks of in vitro culture without the addition of growth factors, we added irradiated PBMCs at the beginning of the third week culture to serve as antigen presenting cells without exogenous allergen specific T cell clones. As IFNγ is secreted by differentiated Th1 CD4+ and cytotoxic CD8+ cells, it serves as a good marker to detect T cell activation in response to Ni allergen. Notably, the IFNγ concentration is much higher by the third week of culture than the first week, indicating enrichment of Ni-specific T cells for both subjects. Under these culture conditions, the percentage of proliferating cells carrying CellTrace fluorescence in the live population is relatively high, 74.1% for P7 and 53.6% for P9.

To analyze the TCR repertoire of these Ni-reactive T cells, the cell ranger pipeline from 10× genomics was used to process Chromium VDJ output, and we analyzed TRAV, TRAJ, TRBV, and TRBJ usage in both CD4+ and CD8+ cells from both subjects. Large-scale paired single cell sequencing of Ni^2+^ specific T cells has not been done before. This technique is being increasingly used to identify pathogenic T cells in many different immune-mediated diseases [28,29]. In this study, we showed several highly expanded T cell clones by sequencing data, and demonstrated the preferential usage of TRBV19, TRBV5-1, and TRBV20-1, consistent with previous studies on Ni^2+^ contact dermatitis [11,16,18,20]. Our study suggests, then, that the certain population of Ni^2+^-reactive T cells in joint implant failure may be shared with the T cells responsible for Ni^2+^ induced contact dermatitis. TRBV19 usage is the highest in the CD4+ population from P9, but not P7. This suggests the genetic background, e.g., MHC, may have different Ni^2+^ binding chemistry and select T cell repertoire. Previous studies have shown the preferential usage of TRBV19 in Ni allergic subjects, and our study confirms its importance. A highly expanded CD4+ clone for P7 is TRAV41-TRBV18, which was detected over 50. We also noted the high percentage of CD8+ T cells in these proliferative populations, accounting for more than 20% of the total sequenced cells. Although previous studies have detected Ni^2+^-reactive CD8+ T cell clones, they did not elucidate the TCR sequences [16,30,31], and our research thus provides important insights into cytotoxic T cell function in Ni allergy. The alignment of CDR3β sequences identified the presence of Glu at the last fourth position of CDR3β, which could be a potential important amino acid in Ni^2+^ coordination. His and Asp/Glu amino acids are common nickel ligands. In our Ani2.3 TCR/DR52c/mimotope structure, Asp and Gln coordinated Ni^2+^ [15]. Interestingly, this Glu is conserved in both CD4+ and CD8+ cells sequenced. The overrepresentation of Glu at similar positions may also imply that there may be common Ni^2+^ presenting self-peptides. In future experiments, we plan to clone these highly expanded T cell clones and identify Ni^2+^ presenting MHC and peptides.

## 4. Materials and Methods

### 4.1. Subject Selection

Subjects were enrolled in an IRB approved study (Study number HS-2511, original approval date is 04 10 2010, most recent approval date is 29 07 2020) at National Jewish Health, and provided informed consent to participate. All samples provided for inclusion in this study were from de-identified subjects and were exempt from IRB review of the University of Colorado. Two patients P7 and P9 were selected for our study on the basis of a positive Ni^2+^ patch test and positive Ni^2+^ LPT, as well as a poorly functioning Ni^2+^ containing implant.

### 4.2. PBMC Culture and Stimulation

PBMC preparation and storage were performed according to previously established methods [18]. In addition, 10^7^ PBMC cells were thawed from P7 and P9 patients, cultured in enriched MEM as previously described [32], and supplied with 10% heat-inactivated premium FBS (VWR, PA, USA, product 97068-085, lot 214B17). Furthermore, 100 μM of NiSO_4_ (Sigma, Germany, product 31483-250G, Lot SZBB0900V) was added to the culture for 7 days. Then, PBMCs were washed twice with BSS, and cultured in media with 20U/mL hIL2 (Sigma, Germany, product 11011456001) for 7 days. Next, PBMCs were washed twice with BSS and returned to media with 100 μM NiSO_4_ and cultured for another 7 days. At this point, PBMCs were stained with CellTrace violet proliferation dye (Invitrogen, City, CA, USA, product C34571) and irradiated PBMCs (40 Gy) from the same patient were added. After 7 days of the second round of Ni^2+^ stimulation, the proliferative cells were sorted based on CellTrace fluorescence.

### 4.3. HLA Genotyping

1 × 10^6^ cryopreserved PBMCs were used to isolate the genomic DNA by using the QIAamp DNA Mini-Kit as previously described [33]. The high resolution HLA-DR typing was performed by ClinImmune Labs (Bioscience 2, Aurora, CO, USA).

### 4.4. IFNγ Measurement

IFNγ was measured using ELISA MAX Standard Set Human IFN-γ (Biolegend #430101).

### 4.5. Cell Staining and Single-Cell Sequencing

The sorted PBMCs were stained based on a Biolegend recommended method for 10× genomics sequencing. The human TruStain FcXTM Fc blocking reagent (Biolegend Cat# 422301) was used to block the Fc receptors, and the PBMCs from P7 and P9 patients were stained with TotalSeqTM-C0252 anti-human Hashtag 2 Antibody (Biolegend Cat# 394663) and TotalSeqTM-C0253 anti-human Hashtag 3 Antibody (Biolegend Cat# 394665), respectively. TotalSeqTM-C0072 anti-human CD4 Antibody (Biolegend Cat# 300567) and TotalSeqTM-C0046 anti-human CD8 Antibody (Biolegend Cat# 344753) were used on both patients to distinguish CD4 and CD8 T cell populations within the sample. After staining, the PBMCs from two patients were merged and 5000 cells were used for single cell V(D)J sequencing by personnel at Genomics and Microarray Core Facility at the University of Colorado Anschutz Medical Campus. There were 40 million reads performed on V(D)J library and 30 million reads on cell hashing/CITE seq library.

### 4.6. Data Processing

The 10× genomics “Cell ranger v3.1.0 vdj” function was used to obtain the paired alpha and beta chain VDJ sequences, and “Cell ranger v3.1.0 count” function was used to analyze gene expression and cell hashing. The gene expression was aligned against the human reference (GRCh38) 3.0.0 dataset.

## 5. Conclusions

In summary, this study characterized the Ni^2+^-reactive T cell repertoires derived from the peripheral blood of joint failure patients using single-cell sequencing techniques. In addition, 2650 out of 5000 productive TCR sequences were identified, and the preferential usage of TCR alpha and beta chain was analyzed. TRBV19 and TRBV20-1 segments are present in a high percentage of both P7 and P9 sequenced T cells, consistent with previous studies on the T cell repertoire from contact dermatitis patients. This suggests that skin and joint sensitization processes could share similar pathogenic T cells. We identified a conserved glutamic acid in the TCR CDR3β sequences in our study, which is a potential nickel binding site. The findings in this study are important for understanding the molecular mechanisms of T-cell mediated nickel hypersensitivity, as well as the development of therapeutics for nickel-induced joint implant failure.

## Figures and Tables

**Table 1:** Summary of clinical data from the two subjects.

Patient	HLA Typing			Joint Replaced	Size of Nickel Patch Test (Range is 1+ to 3+ )	Nickel LPT PSI
7	DRB1*04:01:01, DRB1*07:01P		DRB4*01:BRF	Postop L THA failure	1+	12.2
9	DRB1*07:01P, DRB1*11:01:02	DRB3*02:ANCDJ	DRB4*01:BRF	Postop R TKA failure	3+	51.2

**Table 2:** Most frequent pairs of V-J usage sequenced.

Number of Clones	Percentage	TRAV	TRAJ	TRBV	TRBJ
P7 CD4+					
51	13.49%	TRAV41	TRAJ57	TRBV18	TRBJ1-2
8	2.12%	TRAV16	TRAJ13	TRBV15	TRBJ2-7
4	1.06%	TRAV13-1	TRAJ45	TRBV12-3	TRBJ1-1
3	0.79%	TRAV5	TRAJ22	TRBV5-1	TRBJ2-1
3	0.79%	TRAV17	TRAJ47	TRBV2;TRBV6-4	TRBJ2-6;TRBJ1-1
3	0.79%	TRAV13-1	TRAJ9	TRBV28	TRBJ1-2
2	0.53%	TRAV1-2	TRAJ18	TRBV9	TRBJ2-1
2	0.53%	TRAV19	TRAJ17	TRBV7-3	TRBJ2-2
2	0.53%	TRAV29DV5	TRAJ48	TRBV7-9	TRBJ1-2
2	0.53%	TRAV8-1	TRAJ37	TRBV6-5	TRBJ2-7
P7 CD8+					
9	7.96%	TRAV29DV5	TRAJ56	TRBV6-5	TRBJ1-5
5	4.42%	TRAV34	TRAJ26	TRBV5-1	TRBJ2-7
4	3.54%	TRAV3	TRAJ30	TRBV2	TRBJ2-3
4	3.54%	TRAV41	TRAJ49	TRBV9	TRBJ2-7
3	2.65%	TRAV10	TRAJ12	TRBV19	TRBJ2-1
3	2.65%	TRAV26-2	TRAJ48	TRBV20-1	TRBJ2-7
3	2.65%	TRAV12-2	TRAJ8	TRBV24-1	TRBJ2-5
2	1.77%	TRAV13-1	TRAJ21	TRBV7-3	TRBJ2-5
2	1.77%	TRAV8-4	TRAJ49	TRBV7-9	TRBJ1-1
2	1.77%	TRAV13-1	TRAJ6	TRBV10-1	TRBJ2-7
2	1.77%	TRAV12-3	TRAJ18	TRBV7-9	TRBJ2-4
P9 CD4+					
24	1.47%	TRAV13-1;TRAV23DV6	TRAJ40;TRAJ15	TRBV19	TRBJ1-5
18	1.10%	TRAV23DV6	TRAJ15	TRBV19	TRBJ1-5
12	0.73%	TRAV13-2	TRAJ45	TRBV6-3	TRBJ1-1
11	0.67%	TRAV13-2;TRAV25	TRAJ45;TRAJ37	TRBV6-3	TRBJ1-1
9	0.55%	TRAV39	TRAJ44	TRBV7-2	TRBJ2-3
7	0.43%	TRAV13-1	TRAJ40	TRBV19	TRBJ1-5
6	0.37%	TRAV8-2	TRAJ40	TRBV27	TRBJ2-5
6	0.37%	TRAV9-2	TRAJ31	TRBV27	TRBJ2-7
5	0.31%	TRAV3	TRAJ23	TRBV19	TRBJ1-2
5	0.31%	TRAV9-2	TRAJ57	TRBV14	TRBJ1-1
P9 CD8+					
19	3.97%	TRAV22	TRAJ52	TRBV7-6	TRBJ2-7
9	1.88%	TRAV8-1	TRAJ5	TRBV5-1	TRBJ2-3
8	1.67%	TRAV12-3	TRAJ26	TRBV7-3	TRBJ2-3
6	1.26%	TRAV4	TRAJ20	TRBV24-1	TRBJ2-5
5	1.05%	TRAV27	TRAJ29	TRBV20-1	TRBJ2-1
5	1.05%	TRAV36DV7	TRAJ24	TRBV20-1	TRBJ1-2
5	1.05%	TRAV29DV5	TRAJ33	TRBV7-8	TRBJ1-1
5	1.05%	TRAV13-1	TRAJ32	TRBV12-3	TRBJ2-5
5	1.05%	TRAV21	TRAJ45	TRBV6-5	TRBJ1-5
4	0.84%	TRAV12-1;TRAV20	TRAJ41;TRAJ49	TRBV6-5	TRBJ2-7

## Data Availability

Data available in a publicly accessible repository.

## Abbreviations

PBMC	peripheral blood mononuclear cell
P7	patient #7
P9	patient #9
APC MHC TCR VDJ CDR LPT Postop PSI THA TKA TRAV TRAJ TRBV TRBJ Th1 Tc1	antigen-presenting cell major histocompatibility complex T cell receptor variable (V), diversity (D) and joining (J) gene segments Complementarity-determining region lymphocyte proliferation test Post-operation peak stimulation index Total hip arthroplasty Total knee arthroplasty T-cell receptor alpha variable T-cell receptor alpha J segment T-cell receptor beta variable T-cell receptor beta J segment Th1 helper cell Type 1 CD8+ T cell
